# Facing Sorrow as a Group Unites. Facing Sorrow in a Group Divides

**DOI:** 10.1371/journal.pone.0136750

**Published:** 2015-09-03

**Authors:** Miriam Rennung, Anja S. Göritz

**Affiliations:** Department of Occupational and Consumer Psychology, University of Freiburg, D-79085, Freiburg, Germany; University of Amsterdam, NETHERLANDS

## Abstract

Collective gatherings foster group cohesion through providing occasion for emotional sharing among participants. However, prior studies have failed to disentangle two processes that are involved in emotional sharing: 1) focusing shared attention on the same emotion-eliciting event and 2) actively sharing one’s experiences and disclosing one’s feelings to others. To date, it has remained untested if shared attention influences group cohesion independent of active emotional sharing. Our experiment investigated the effect of shared versus individual attention on cohesion in groups of strangers. We predicted that differences in group cohesion as called forth by shared vs. individual attention are most pronounced when experiencing highly arousing negative affect, in that the act of experiencing intensely negative affect with others buffers negative affect’s otherwise detrimental effect on group cohesion. Two-hundred sixteen participants were assembled in groups of 3 to 4 people to either watch an emotion-eliciting film simultaneously on a common screen or to watch the same emotion-eliciting film clip on a laptop in front of each group member using earphones. The film clips were chosen to elicit either highly arousing negative affect or one of three other affective states representing the other poles in Russel’s Circumplex model of affect. We examined self-reported affective and cognitive group cohesion and a behavioral measure of group cohesion. Results support our buffer-hypothesis, in that experiencing intense negative affect in unison leads to higher levels of group cohesion than experiencing this affect individually despite the group setting. The present study demonstrates that shared attention to intense negative emotional stimuli affects group cohesion independently of active emotional sharing.

## Introduction

During collective gatherings, ceremonies or political demonstrations group emotions abound. While research on individually experienced emotions has a long tradition, group emotions and how they relate to individually experienced emotions remain poorly understood (for a review, see [1]). Likewise, researchers who have investigated the bonding effect of interpersonal synchronization noted that the effects of different forms of synchronization (e.g., behavioral, perceptual, emotional) have remained understudied [2]. However, group emotions, also referred to as emotional sharing or emotional synchrony, seem to fulfill important social functions [3–6], the most prominent being bonding [6]. Building on the seminal work of Durkheim [7], a recent study revealed that perceived emotional synchrony with others mediates the strengthening of social bonds in collective gatherings [8]. Gaining a better understanding of the social effects of group emotions, and above all of negative emotions, is crucial for effectively coping in times of crisis. Specifically, it is worth investigating if experiencing negative emotions individually compared to sharing the negative experience with the group makes a difference for social bonding. An example is the practical and sometimes managerial question of how to communicate bad news in groups.

Sometimes, the context in which bad news are broken is unnecessarily ill-chosen: Radio Shack as well as Dell Computers informed lay-off victims via e-mail of their fate without further information [9,10] notwithstanding ample evidence that the way bad news such as downsizing are delivered has a substantial impact upon their reception [10].

There is much to suggest that the social consequences of experiencing negative emotions are indeed contingent on whether negative emotions are experienced individually or in a group: Sharing negative affect with others, be it after a tragic event [11] or induced in the laboratory [12] was found to increase social bonds, while inducing negative affect individually decreased social identification and enhanced personal identity salience [13].

However, emotional sharing as referred to in prior studies usually confounds two processes: a) jointly focusing attention on the same emotion-eliciting stimuli and b) actively sharing the emotional experience with others by talking about it or expressing it through overt action (yelling, singing, dancing etc.) (see [8] for a discussion of the multifaceted process underlying emotional synchronization). It is unclear if the mere assumption that other people are experiencing the same affect (i.e., shared attention) suffices to heighten social cohesion or whether the emotional experience needs to be actively shared. At this point it cannot be excluded that it is the act of speaking about negative experiences or disclosing one’s feelings to others otherwise that accounts for the differences in connectedness as a function of individual versus shared emotional experiences (for a review on active emotional sharing see [14]). To address the question whether merely sharing attention to emotional content suffices to heighten social cohesion, in this study we deliberately manipulated attention (shared versus individual) while withholding the opportunity to actively share the emotional experience.

In accordance with prior research on group emotion [15], we apply a broad definition of affect as a subjective feeling state [16] that can range from diffuse moods to intense emotions. Thus, in this paper we will use the terms mood, affect and emotion interchangeably.

As dependent variable we chose to assess group cohesion, because it captures best the overlap of dependent variables investigated in prior studies. Although historically group cohesion was defined as a single force, *interpersonal attraction*, it is now understood as a multifaceted construct [17]. Bollen and Hoyle [18] differentiate two components 1) *sense of belonging* and 2) *morale*. Sense of belonging pertains to a cognitive sense of identification, whereas morale represents the affective component reflecting a global affective response. We deemed this approach most useful for the purpose of this study, as the two components are applicable to a broad range of groups, including those with no prior interaction [17].

In the following paragraphs we outline our predictions concerning the social effect of shared versus individual attention as well as the role of type of affect, and we present empirical evidence to support our predictions.

### Shared Attention

Evidence is mounting that emotional sharing elicits or strengthens social ties [8,19,11,12]. Here, we argue that actively sharing an emotional experience may not be necessary for cohesion to evolve. We expect that focusing one’s attention on the same emotional stimulus as those people around oneself and being aware of this shared focus is sufficient to spark connectedness. Due to more subtle cues as to companions’ emotional states when merely sharing attention to emotional material as compared to stronger cues when actively sharing one’s emotional state (e.g., words, yelling, movement) we think it likely that effects of merely sharing attention to emotional content be smaller than effects of active emotional sharing. Although cues as to companions’ emotional states may be subtle or virtually absent when sharing attention to the same event, paralleling findings in the domain of attitudes, people are likely to assume alignment of their own affective reaction with the affective reaction of those around them (i.e., false consensus effect, see [20]). In other words, in the absence of visible cues, people tend to believe that others think and feel as they do, thus inferring (sometimes mistakenly) affective synchrony. Importantly, it is essential that people *infer* synchronous experience; it is not necessary that the other person actually feels the same for closeness to prosper [21]. Thus, being exposed to the same emotional stimulus at the same time might spark social bonding just as active emotional sharing, as people tend to assume emotional synchrony. In line with this reasoning, Paladino, Mazzurega, Pavani, and Schubert [22] showed that mere synchronous multisensory stimulation leads to feelings of unity. Specifically, these researchers brushed the cheek of participants, while at the same time the participants saw the face of a confederate on a computer screen either being brushed synchronously or asynchronously. Participants in the synchronous condition felt significantly more similar and closer to the confederate, experienced more self-other overlap, and showed more conforming behavior. In other words, synchronous stimulation (without talking about the experience) elicits a feeling of oneness with the other person or “identity fusion” as Swann, Jetten Gómez, Whitehouse, and Bastian [19] refer to it.

When studying the effect of shared attention to an emotional event, the role of the type of affect is of interest. In the current experiment, we were particularly interested in the role of negative affect and compared its effect to a compilation of other emotional states. On the one hand, one might intuitively assume highly positive emotions to be more potent in fostering cohesion than negative emotions, and Rimé reviews some evidence supporting the view that sharing positive emotions can act as social glue, too [14]. On the other hand, the seminal work by Schachter on the anxiety-affiliation link [23] suggests that people seek the company of others especially in stressful situations. Thereby, negative rather than positive affect, seems to motivate people to bond with other people—especially with those who are in a similar plight. This early research is supplemented by more recent evidence, which suggests that shared experiences are more powerful in eliciting a feeling of connectedness when they are negative rather than positive [24]. In the domain of rituals, field and laboratory studies indicate that intense, negative practices are specifically powerful in eliciting cohesion among participants [25–27].

Apart from the valence of affect, arousal is the second dimension on which affective states can vary [28]. Research on relationships shows that participating in arousing activities enhances relationship quality [29], thus suggesting that high arousal contributes to social bonding more than low arousal.

Neuroimaging studies buttress these finding concerning the role of valence and arousal for interpersonal processes. Nummenmaa and colleagues [30] found evidence that watching emotionally laden movie clips synchronizes brain activity across participants, and they specifically looked for the type of emotions that best predict intersubject correlation (i.e., correlation of fMRI data). They observed that negative valence increased intersubject correlation for activation of the default-mode network, which is linked to social cognitive processes [31], whereas high arousal synchronized activation in the somatosensory cortices, which play a major role in understanding others’ actions. It was repeatedly shown that observing the behavior and bodily sensations of others elicits vicarious activation in these brain areas [32]. More specifically, experiencing negative affect leads to a matching of brain activation and triggers brain areas that are crucial for self-other interaction. High arousal sets the mental ground for slipping into another person’s shoes by directing attention to similar features of the environment [30]. These findings were conceptually replicated for auditory stimuli, that is, listening to an emotional speech [33]. Summing up, the synchronous experience of negative and arousing affect puts us in a position to understand other’s actions and lays the ground for social bonding. In accordance with this body of research, we predict that in the shared attention condition, highly arousing negative stimuli elicit the highest level of social cohesion.

### Individual Attention

Based on prior research we expect that attending to negative stimuli individually will have different effects on social cohesion than sharing attention with others. It was found that experiencing negative affect alone is detrimental to social identification: In a study on call center employees the induction of negative affect as compared to positive affect decreased ratings of organizational identification [13]. The rational for mood effects stems from two related lines: (1) mood-congruency [34] refers to the indirect effect of mood due to the heightened accessibility of mood-congruent concepts; (2) affect-as-information [35] presumes a direct mood effect in that people use their current feelings as a source of information—even if these feelings are unrelated to the object under consideration (e.g., even if mood is experimentally induced and the correct source of affect is known to participants, [36–38]. Forgas [37] integrates these mechanisms in his Affect Infusion Model and elaborates under which conditions affect is most likely to influence judgment. He reviews evidence showing that affect impacts judgment most (1) on constructive tasks such as social judgments [39], (2) if no detailed information is available to make a more informed choice and (3) if the decision is of little personal relevance [40]. All of these conditions apply in the current experiment: After the experimental manipulation, participants were asked to judge how close they feel to their co-participants whom they had never met before, and therefore (1) the social judgment had to be constructed on the spot rather than retrieved from memory, (2) participants had no further information about their fellow group members, and (3) participants’ judgment was of little personal relevance because it was of no consequence. In line with the Affect Infusion Model, research in the field of social cognition found that social judgments are substantially affected by emotional states in that social stimuli are generally evaluated in a mood-congruent manner [41], resulting in a more favorable evaluation of a social group when one is in a positive mood and a more negative evaluation when being in a negative mood (e.g., [42,43]). There is ample evidence that the experimental induction of positive mood in an individual setting fosters social bonding: Positive mood was shown to facilitate sociality [44], self-other overlap [45], compassion [46], interpersonal trust [47] and prosocial behavior [48].

Regarding arousal, a major review found that the relationship between arousal and valence is best represented by a V-shaped curve [49], which is in line with the assumption that arousal reflects the intensity of pleasure or displeasure [50–52]. Based on this evidence, we predict that arousal amplifies mood-congruent judgment. Consequently differences between negative and positive mood on social cohesion will be most pronounced when arousal is high. Therefore, in the individual attention condition, we predict that participants will feel the lowest level of social cohesion when in a highly arousing negative mood.

### Hypothesis

Summing up, we expect that the shared and individual attention condition will differ when highly arousing negative affect is induced: In the individual attention condition we expect mood-congruency, that is, given the absence of more relevant information concerning their connectedness to the group, participants rely on their mood to make a decision about their level of connectedness. By contrast, in the shared attention condition, judgments concerning social connectedness are informed by the previously shared experience, whereby co-experiencing highly negative mood makes people bond more than co-experiencing non-negative mood.

Thus we propose a buffer-hypothesis: Experiencing intense negative affect in unison mitigates the otherwise cohesion-decreasing effect of individually experiencing negative affect. We will put these predictions to the test by comparing the effect of highly arousing negative affect with three other types of affect that systematically vary in valence and arousal.

The study at hand has three goals: (1) to investigate whether shared attention to an emotional stimulus has similar cohesion-enhancing effects as active emotional sharing. To our knowledge, no experiment has illuminated the effect of shared attention on group cohesion yet, and thus, we answer the call to try to better understand the complex effects of interpersonal synchrony on human sociality as put forward by Gill [2]; (2) to examine whether the effect of shared attention is contingent on the type of affect shared. Given the hitherto evidence, we hypothesize that shared attention to highly arousing negative rather than positive affect or low arousal, fosters group cohesion in participants compared to individual attention; (3) to gain a more thorough understanding of which aspects of cohesion, if any, are influenced by shared attention. To this end, we comprehensively measure group cohesion both on an affective as well as a cognitive level. Furthermore, with similarity we measure a pre-cursor of social cohesion [53]. Moreover, we include self-reports as well as a behavioral measure to find out if shared attention has real-life behavioral consequences besides its possible effects on mental processes. As we measure several facets of cohesion we explore the causal relationships among these facets using mediation analyses.

## Methods

### Ethics Statement

All participants volunteered and provided a written informed consent. When planning, advertising, and conducting the study, we conformed to the German Psychological Society (DGPs) ethical guidelines (2004, CIII) as well as APA’s ethical standards. Ethics approval was obtained from the German Psychological Society (AG 062013). All data were collected and analyzed anonymously.

### Participants and Design

Two-hundred sixteen students of psychology (30.6%), education (24.5%), natural sciences (10.6%), humanities (8.3%), law (8.3%), economics (6.5%), social sciences (6.0%), and medical science (4.6%) participated in the study. Students participated in groups of three to four people. We employed a 2 (attention: shared vs. individual) x 4 (film clip: high arousal/negative valence vs. high arousal/positive valence vs. low arousal/negative valence vs. low arousal/positive valence) between-subjects design, which resulted in eight experimental conditions. The groups were randomly assigned to one of the eight conditions. Participants received on average 7.50€ as compensation. The exact amount was decided through a raffle at the end of the study, with the compensation ranging from 2.50€ to 12.50€, as it was important for one of the outcome measures that participants did not know their exact compensation in advance.

### Operationalization and Procedure

#### Attention

Participants were seated in a semicircle of tables facing the center of the semicircle. In the shared-attention group, participants watched a film clip together on a big screen in the center of the semicircle. In the individual-attention group, participants watched the film clip individually on a laptop on the table in front of them using earphones. In the individual attention group, participants were told that the film clip was assigned at random and that their co-participants might watch the same or a different film clip on their laptop. The tables and laptops were arranged in a way that did not allow participants to see the screen of their co-participants’ laptops. Thus, participants in the individual attention group did not assume that their entire group was exposed to the same clip. In reality, all participants in group watched the same film clip, and thus the same emotion was induced.

#### Affect

Participants were exposed to short film clips that lasted on average 4 minutes. We contrasted a film clip inducing high-arousal negative affect with three clips that were complimentary in terms of valence and arousal, representing the other ends in Russel’s Circumplex model [28]. This allowed us to compare the effect of highly arousing negative affect to a comprehensive array of other affective states. We selected the film clips based on Schaefer, Nils, Sanchez, and Philippot [54], who developed a database of emotional film clips and computed scores for valence and arousal based on 364 participants' ratings. We compiled four film clips: The high arousal/negative affect clip included a scene from the movie “Schindler’s List” that showed dead bodies being carried away in a concentration camp and a scene about sexual abuse of children. The low arousal/negative affect clip showed dull situations eliciting a bleakish atmosphere. The low arousal/positive affect clip included a comical scene taken from the movie “Benny and Joon” as well as a scene depicting a harmonious interaction between two lovers. The high arousal/positive affect clip included a scene from the movie “Dead Poets Society” in which students express their support for a former teacher, and it included a scene from the movie “Life is Beautiful” in which a father and a boy in a concentration camp talk to the mother using a loud speaker which sounds reached the whole camp.

#### Procedure

When participants arrived at the laboratory, they were seated in front of one of the four tables (either fitted with a laptop or empty) arranged in a semicircle. Participants were told that the aim of the study was to investigate the link between personality traits and the evaluation of corporate designs. They were led to believe that each group was assigned a unique corporate design and would evaluate it in the course of the study. In reality, all groups saw the design of the company “Salt + Pepper”. Salt + Pepper is a small service company that agreed to support our study by sponsoring merchandising objects that come in their corporate design. To establish the corporate design as a symbol that distinguished participants’ own group from other groups, objects in different corporate designs were placed on a table within the sight of the participants. Thus, participants assumed that their group was the only one who was allotted the design of the Salt + Pepper company while thinking that the other groups participating in the experiment would evaluate another design. Next, participants were asked to imagine that they were employees of Salt + Pepper. They were asked to write their name on a tag in a Salt + Pepper corporate design and to attach it to their shirt. This procedure provided participants with a marker of group membership in order to generate common identity. In supporting the generation of common identity in all participants, we were able to explore whether the between-subjects manipulations increased group cohesion beyond the effects of common identity, which is an established source of group cohesion (e.g., [55]). For a similar procedure see [56]. After that, participants were asked to indicate their current mood using the Self-Assessment Manikin (SAM, [57]). Thereafter, the participants watched one out of four 4-minute film clips, either individually or as a group. Next, the participants filled in a questionnaire containing the SAM for measuring postfilm affect, the group cohesion and similarity scales, and socio-demographic questions. Subsequently, participants were exposed to three objects in the Salt + Pepper corporate design (a mouse pad, a water bottle, and a cup), and they stated how much they appreciated this corporate design using 11 items (see materials). Afterwards, the participants were told that they had the chance to buy the Salt + Pepper cup whose corporate design they had just evaluated. Participants were asked to write down how much they were willing to pay for the cup. They were informed that if the amount they wrote down was above a threshold set in advance that was unknown to the participant, the transaction would take place for real, and if their offered amount was under the threshold, the transaction would not take place. In case of a transaction taking place, the amount the participants had written down to pay would be subtracted from their later compensation for participation. The amount of compensation paid for participation in the study was determined through a raffle at the end of the study with an average payout of 7.50€. Consequently, the amount the participants chose to pay for the cup was not contingent on their particular compensation. When participants had made their written offer, the experimenter revealed to the participants whether the price they were willing to pay was above the preset threshold, and hence it was revealed if they had actually bought the cup or not. Finally, the participants were probed for suspicion, and they were thanked for participating in the study, paid and debriefed. To ensure that effects are not distorted by unintended interactions between participants, the following steps were taken to reduce verbal contact among participants before and during the study: 1) There was no waiting area, so that participants entered the lab directly. 2) Immediately after arrival, participants were given the consent form to read and sign so that they were busy reading and had no time to talk to their co-participants. 3) Participants were given no opportunity to talk about the film clip during the study, but were given the questionnaire to work on immediately after watching the film clip.

### Materials

#### Affect

We used the Self-Assessment Manikin (SAM) to assess the participants’ emotional state prior to and after watching the film clip. The SAM is a visual measure containing a 9-point scale for valence of affect and a 9-point scale for arousal [57]. On the valence dimension, the scale ranged from 1 = “unhappy” to 9 = “happy”; on the arousal dimension the scale ranged from 1 = “calm” to 9 = “excited”.

#### Group cohesion and similarity

Due to the lack of research on the link between shared attention and group cohesion, we used a comprehensive exploratory approach and additionally assessed perceived similarity, which is an antecedent to cohesion.

*Perceived similarity* contained one item adapted from the Group Identification subscale “In-group Ties” [58] and one item adapted from Wiltermuth and Heath [56] (e.g., “I have a lot in common with the other participants”). With regard to cognitive and affective aspects of group cohesion, *cognitive representation of social closeness* included the visual other-in-self-scale [59] that assesses perceived group entitativity, three items adapted from the group identification subscale “In-group Ties”, three items adapted from the Group Cohesiveness Scale [60], one item adapted from the Emotional Solidarity Scale [61], as well as one item adapted from Wiltermuth and Heath [56] (e.g., “I feel strong ties with other participants”), while *pleasant sentiments towards the group* was assessed via the Group Identification subscale “In-group affect” [58], as well as two items adapted from the Emotional Solidarity Scale [61], and one item adapted from Wiltermuth and Heath [56] (e.g., “I trust the other participants”). We translated the scales into German using the collaborative and iterative translation method [62]. We cooperated with two experienced translators who provided the back translation. We discussed the results in a team of three experts and pretested the pilot questionnaire on a group of 30 students. After data collection we submitted the group cohesion and similarity items to an exploratory factor analysis to test if the assumed differentiation between similarity and group cohesion, as well as the subdivision of the scale in a cognitive and an affective component is supported by the data (for the final group cohesion scale see Table 1).

**Table 1:** Scales and items.

Similarity	Closeness & Liking	Liking of Group Symbol
1. I have a lot in common with the other participants.	1. I identify with the other participants.	1. dull—energetic
2. I am similar to the other participants.	2. I feel affection towards the other participants.	2. long-known—innovative
	3. I feel strong ties to the other participants.	3. inexperienced—experienced
	4. In general, I’m glad to be a member of this group of participants	4. amateurish—professional
	5. I feel I am on the same team with the other participants.	5. unappealing—appealing
	6. I trust the other participants.	6. dubious—reputable
	7. Inclusion of Other in Self Scale [59]*	7. not credible—credible
		8. boring—interesting
		9. In general, do you like the design? (not at all—a lot)
		10. Imagine you were looking for a job and had the appropriate qualifications: would you like to apply for a position in this company? (surely not—absolutely)
		11. Would you like to learn more about this company? (surely not—absolutely)

*Note*. For Similarity as well as Closeness & Liking we used a 7-point Likert scale (except for item 7), for Liking of Group Symbol we used a 9-point semantic differential anchored by the two adjectives displayed in this table (Item 1–8) or a 9-point Likert scale anchored as indicated in parentheses (Item 9–11), respectively.

* We adapted the visual 6-point scale to fit our study by using diagrams displaying only four instead of five circles that move increasingly closer to each other as the diagrams progress from the first to the sixth.

#### Appreciation of group symbol

As people tend to attach a higher value to groups they identify with (for a review on in-group bias see [63]), we additionally assessed the liking of an object that symbolized the ad hoc group. We measured participants’ liking of the group symbol (i.e., the Salt + Pepper corporate design) using a self-report and a behavioral measure: With regard to the self-report, three objects in the corporate design were presented (i.e., a mouse pad, a water bottle, and a cup), and participants rated how much they liked the corporate design as manifested in the three objects using 11 items framed as semantic differentials (e.g., “innovative vs. long-known”). Like the cohesion and similarity items, the liking items were submitted to an exploratory factor analysis (for the final liking scale see Table 1). With regard to the behavioral measure, participants wrote down the amount of money they were willing to pay for the cup featuring the corporate design (i.e., the group symbol). With Appreciation of Group Symbol we complemented the proximal measures (i.e., Similarity and Closeness and Liking) with more distal measures of cohesion, thus tapping behavioral consequences of group identification.

### Preliminary Analyses

Based on statistical item analyses of the original 17-item scale reflecting group cohesion and similarity, items with inadequate discriminatory power, difficulty, and homogeneity were removed. The resulting modified scale had nine items. An exploratory principal axis factor analysis was conducted on the nine items with oblique rotation (direct oblimin). We chose oblique rotation, as we expected the dimensions underlying the cohesion and similarity measure to be correlated. The factor analysis justified the retention of two factors. The items that clustered on the same factor suggested that Factor 1 represented cognitive and affective facets of cohesion (i.e., closeness and liking), while Factor 2 represented similarity as an antecedent to cohesion. Both subscales had good reliability: closeness and liking: Cronbach’s α = .80; and similarity: Cronbach’s α = .79. Furthermore, based on statistical item analyses pertaining to the liking of the group symbol, all 11 items were retained because of adequate discriminatory power, difficulty, and homogeneity. Based on a principal axis factor analysis with oblique rotation (direct oblimin), we opted for a one-factor solution. The scale showed high reliability: Cronbach’s α = .85.

## Results

### Participants

Analyses were based on 198 participants due to the exclusion of 18 participants: Four were excluded because their group consisted of only two participants due to no-shows, nine were excluded because they reported to know at least one of the other participants in their group, and five were excluded because they guessed the hypothesis of the study (i.e., watching the film clips together was meant to make them feel closer to the other participants).

### Prefilm Mood

To ensure that the potential effects of the manipulation were not due to prior mood differences, we tested if the participants in the eight conditions (i.e., four different film clips attended to together or individually) differed in prefilm mood. We ran two one-way ANOVAs with condition at eight levels as independent variable and prefilm self-reports of valence or arousal of affect, respectively, as dependent variables. Using Pillai’s trace there was no significant effect of condition on prefilm arousal, F(7, 189) = 0.89, *p* = .52, η_P_^2^ = .03, or valence, F(7, 189) = 1.86, *p* = .08, η_P_^2^ = .06. However, as the *p*-value for valence is only marginally above the threshold for significance, we decided to include prefilm valence as a covariate in the manipulation check.

### Manipulation Check

We conducted a two-way MANCOVA of film clip and attention on postfilm valence and arousal including prefilm valence as covariate. Thereby we tested if our mood induction was successful and if it worked equally well in the two attention groups. Using Pillai’s trace, there was a significant main effect of film clip on self-reported valence and arousal, F(6, 376) = 16.58, *p* < .001, η_P_^2^ = .21, while neither the main effect of attention, F(2, 187) = 2, *p* = .14, η_P_^2^ = .02, nor the attention x film clip interaction was significant, F(6, 376) = 0.61, *p* = .73, η_P_^2^ = .01. This indicates that the film clips influenced self-reported mood successfully and that neither mood nor the effect of film clip on mood was affected by attention. Subsequent univariate ANOVAS indicate that film clip significantly affected both self-reported valence, F(3, 188) = 42.8, *p* < .001, η_P_^2^ = .41, and self-reported arousal, F(3, 188) = 4.04, *p* = .008, η_P_^2^ = .06

**Fig 1:**
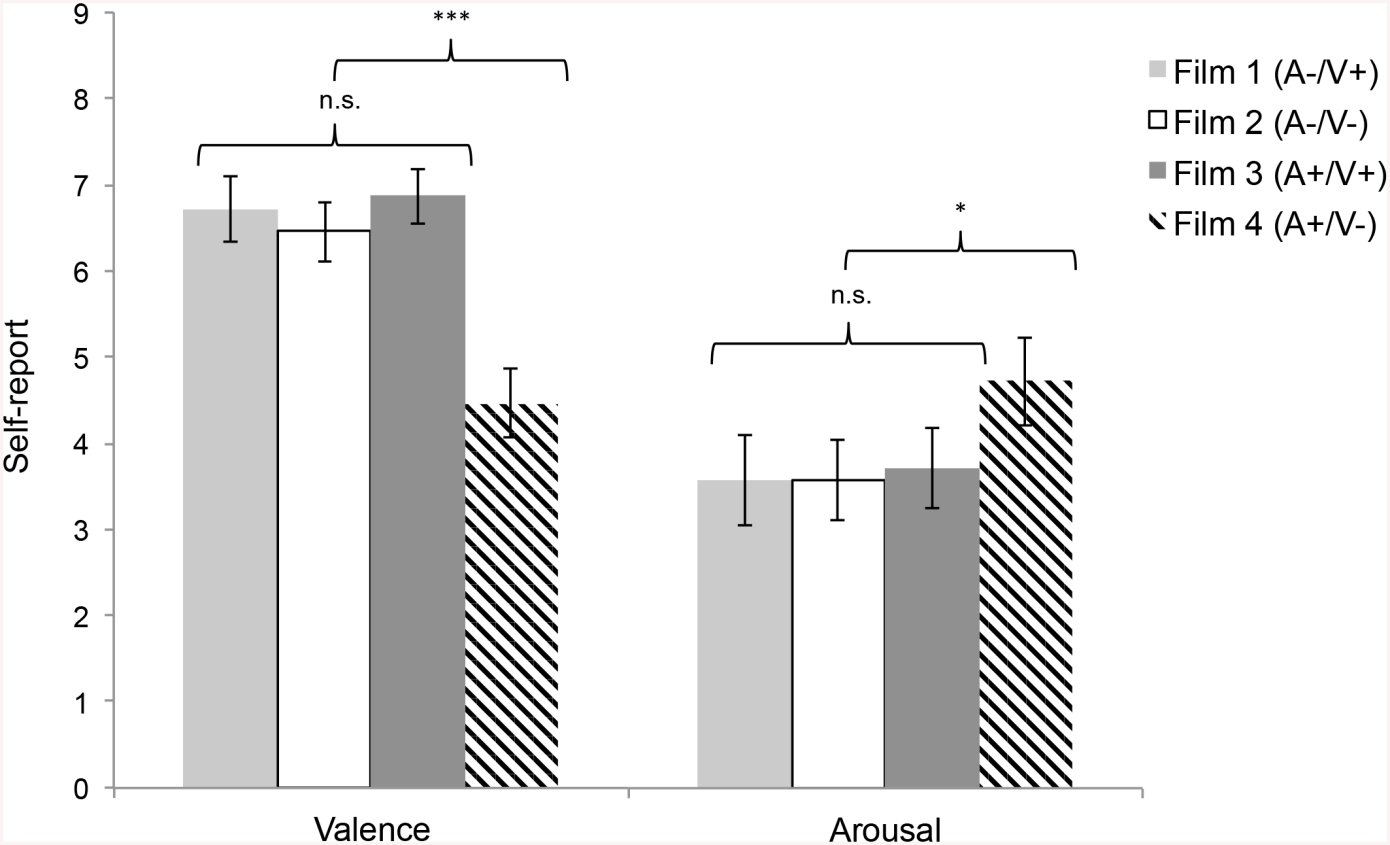
Manipulation Check. Self-reported valence and arousal with the four film clips.

Post-hoc tests using the Games-Howell procedure revealed that compared to the other three film clips, the high arousal, negative film clip led to more negative self-reported valence of affect, all *p* < .001, and higher self-reported arousal compared to the other three film clips, all *p* < .03. The other three film clips did not differ in terms of self-reported valence, all *p* > .3, or arousal, all *p* > .9 (Fig 1). Those who watched the highly arousing negative affect film clip reported an average valence of affect of 4.46 on the 9-point Likert scale ranging from “unhappy” to “happy” and an average arousal of 4.72 on the 9-point Likert scale ranging from “calm” to “exited”. By contrast, those who watched one of the other three clips reported an average valence of 6.68 and an average arousal of 3.61, indicating that they felt rather happy and calm. These results connect to research on the Circumplex model of affect showing that valence and arousal are co-related in that high arousal intensifies valence [50–52] as well as research on mood induction that shows that negative mood is more successfully induced than positive mood (e.g., [64]). Additionally, due to the nonlinear and nontrivial nomothetic relation and the unlawful ideographic relation between valence and arousal [49], Kuppens et al. recommend considering the idiographic properties of the sample when investigating the effect of affect on psychological phenomena.

Therefore, we simplified our focus on mood and collapsed the three film clips that did not differ in self-reported valence and arousal and compared them to the high arousal, negative film clip. Hence, all further analyses were conducted comparing the “negative mood group” to the “non-negative mood group”.

### Main Analysis

A 2 (mood: negative vs. non-negative) x 2 (attention: shared vs. individual) MANOVA was performed on four dependent variables reflecting different facets of group cohesion: (1) similarity, (2) closeness and liking, (3) liking of group symbol, and (4) amount paid for object featuring the group symbol (Table 1). SPSS GLM was used for the analyses with adjustment for unequal cell sizes. There were no univariate or multivariate within-cell outliers at *p* < .001. Results of evaluation of assumptions of normality, homogeneity of variance-covariance matrices, and linearity were satisfactory.

**Fig 2:**
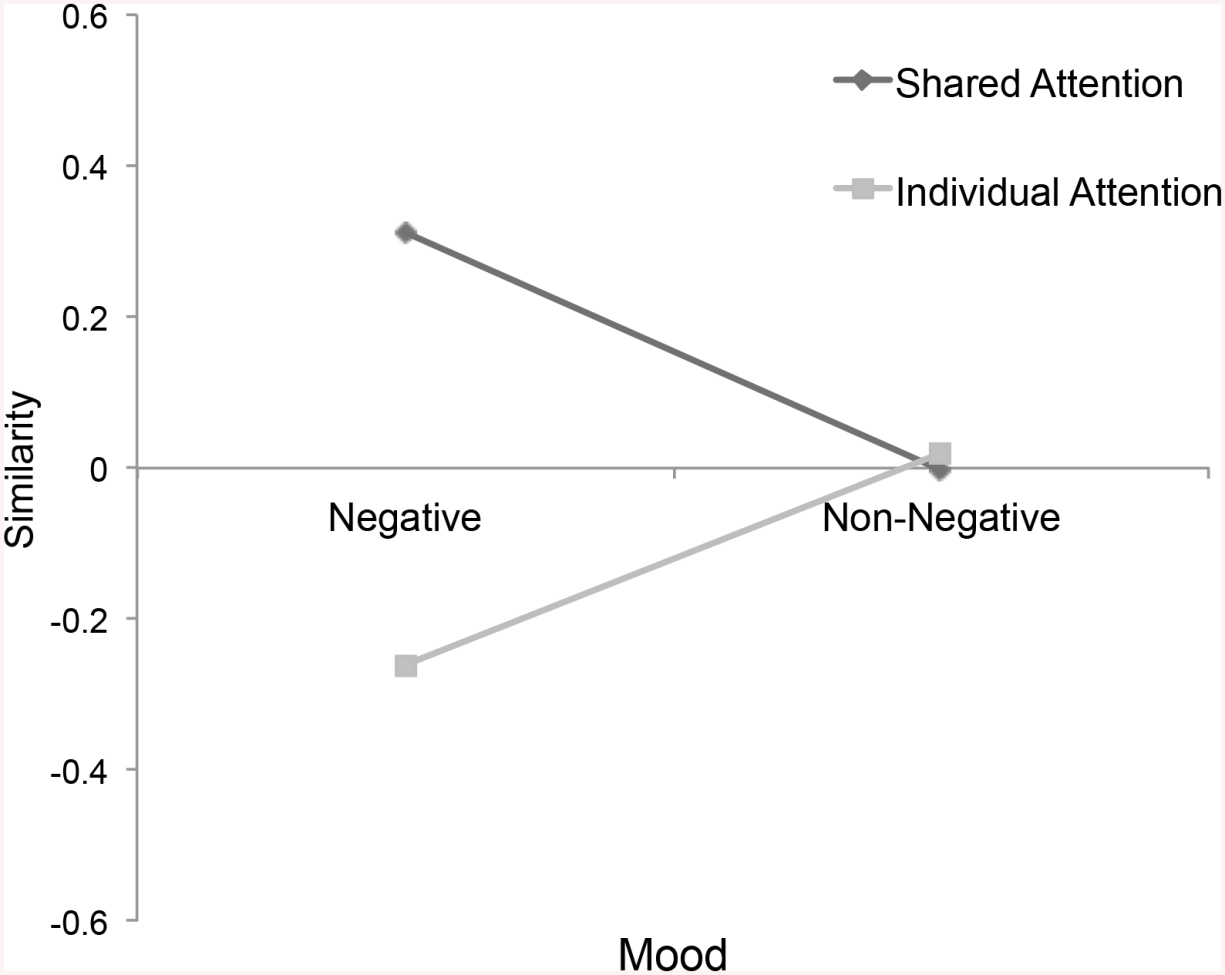
Similarity. Self-reported similarity by attention and mood.

**Fig 3:**
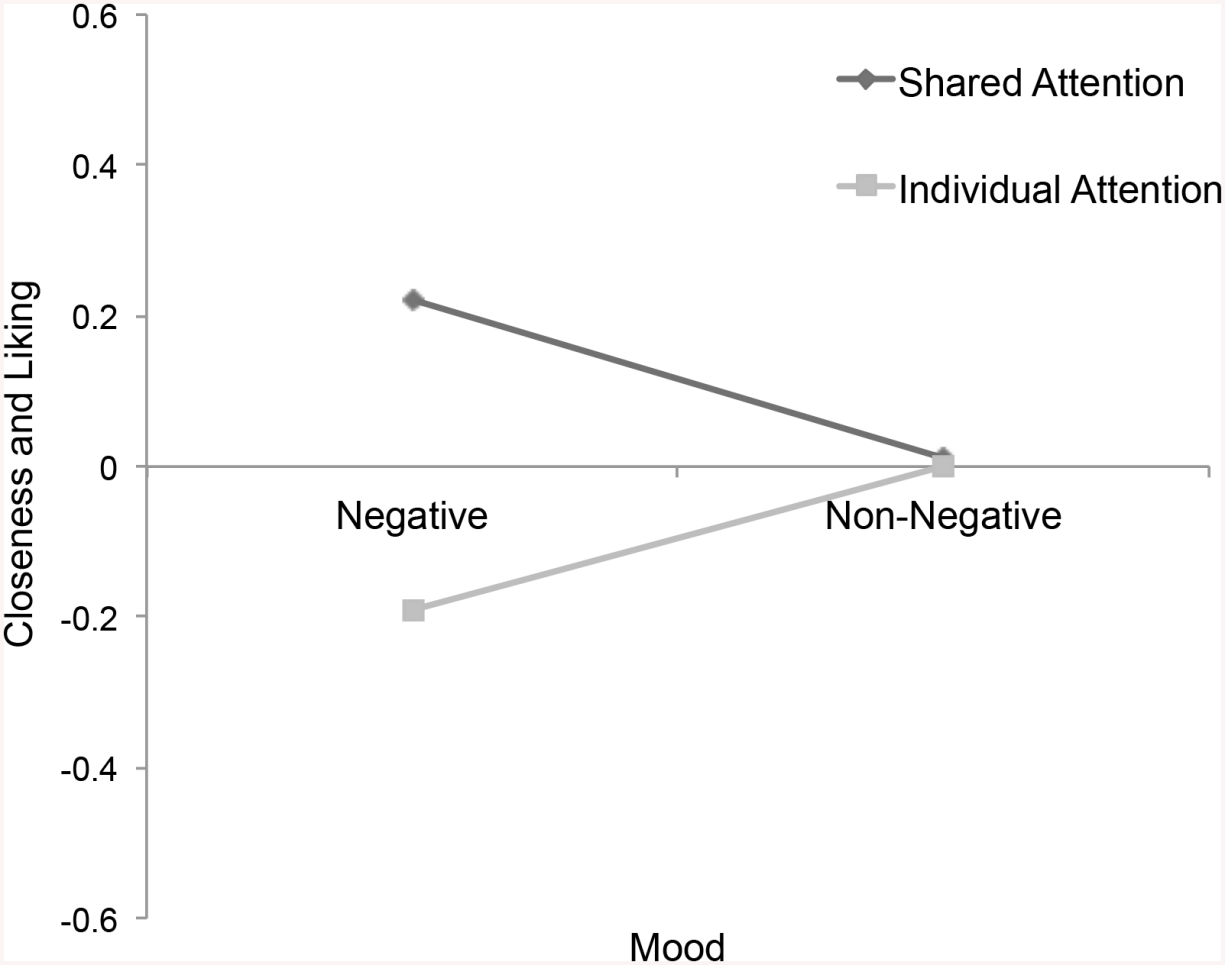
Closeness & Liking. Self-reported closeness and liking by attention and mood.

**Fig 4:**
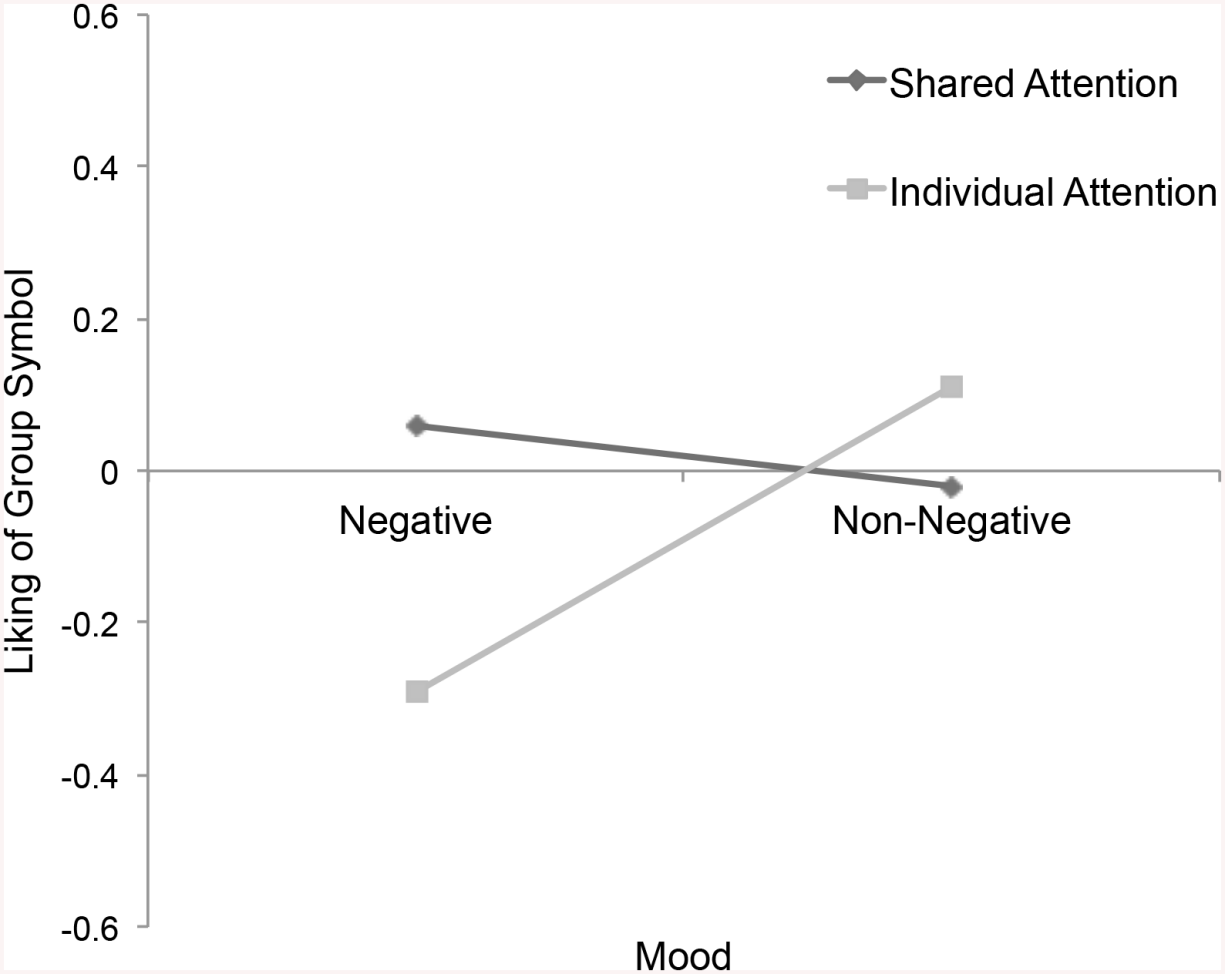
Liking of Group Symbol. Self-reported liking of group symbol by attention and mood.

**Fig 5:**
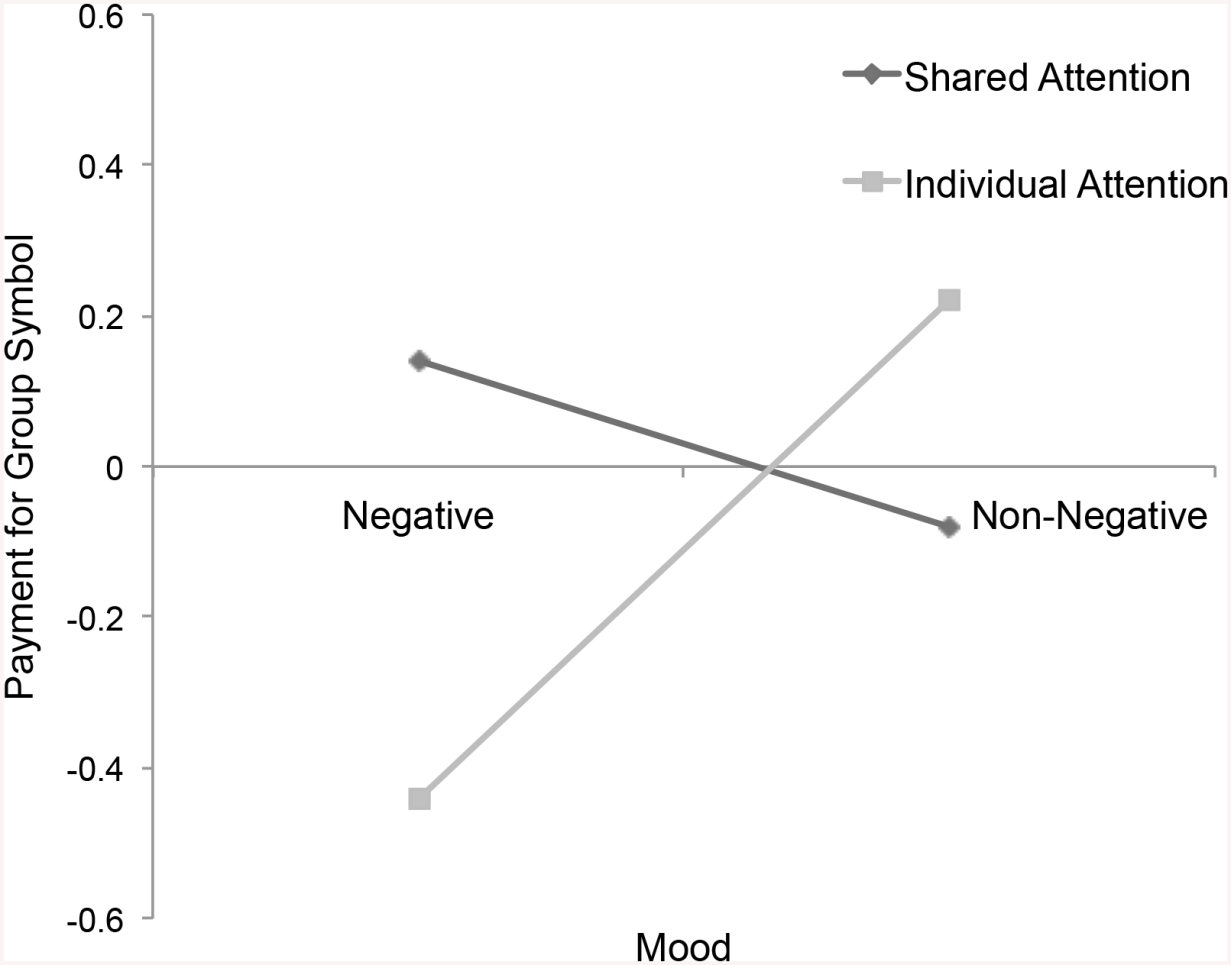
Payment for Group Symbol. Payment for group symbol by attention and mood.

Using Pillai’s trace, there was a significant mood x attention interaction on the four dependent variables, F(4, 191) = 3.29, *p* = .01, η_P_^2^ = .06. That is, type of attention (shared vs. individual) affected cohesion differently depending on mood. Multivariate simple effects analysis revealed that with negative mood, attention significantly affected the four dependent variables, F(4, 191) = 2.67, *p* = .03, η_P_^2^ = .05. In the non-negative mood condition no difference between the individual and shared attention group emerged, F(4, 191) = 1.13, *p* = .34, η_P_^2^ = .02. The follow-up univariate simple effects analysis indicated a similar pattern for all four dependent variables after synchronously experiencing negative mood: Participants felt more similar, *p* = .04, η_P_^2^ = .02, reported higher levels of closeness and liking, *p* = .04, η_P_^2^ = .02, liked the group symbol more, *p* = .056, η_P_^2^ = .02, and were willing to pay more for the group symbol, *p* = .049, η_P_^2^ = .02, than participants who had experienced negative mood individually (Table 2 and Figs 2–5).

**Table 2:** Means and standard deviations of dependent variables by attention and mood.

	Shared Attention		Individual Attention	
	negative mood	non-negative mood	negative mood	non-negative mood
Similarity	0.31 (0.86)	-0.002 (0.85)	-0.26 (0.86)	0.02 (0.98)
Closeness & Liking	0.22 (0.52)	0.01 (0.61)	-0.19 (0.66)	0.002 (0.78)
Liking of Group Symbol	0.06 (0.58)	-0.02 (0.60)	-0.29 (0.67)	0.11 (0.63)
Payment for Group Symbol	0.14 (1.27)	-0.08 (0.93)	-0.44 (0.51)	0.22 (1.08)

*Note*. Means are displayed for each experimental group for each dependent variable with standard deviations in parentheses. For all dependent variables items were z-standardized before calculating the mean.

To examine the relationship between the four dependent variables, we looked at their correlations. As expected, the highest correlations were observed among the two proximal (Similarity and Closeness & Liking) and the two distal measures (Liking of Group Symbol and Payment for Group Symbol), respectively (Table 3), indicating that they tapped two distinct facets of group cohesion.

**Table 3:** Correlation between the four dependent variables.

	Similarity	Closeness & Liking	Liking of Group Symbol	Payment for Group Symbol
Similarity	1	.48**	.30*	.23
Closeness & Liking	.48**	1	.03	.26
Liking of Group Symbol	.30*	.03	1	.47**
Payment for Group Symbol	.23	.26	.47**	1

*Note*. Pearson’s two-tailed correlations are displayed for the negative-mood condition (N = 46).

** *p* < .01,

* *p* < .05.

**Fig 6:**
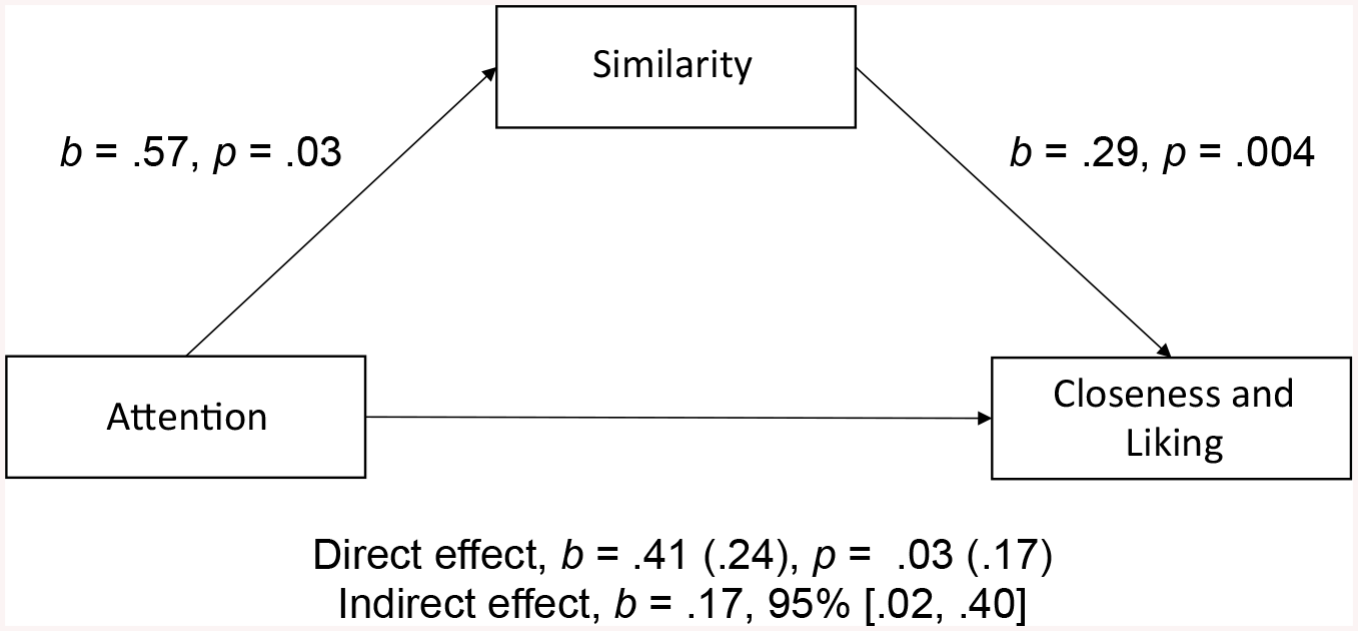
Similarity as mediator. Model of Attention as predictor of Closeness & Liking, mediated by Similarity. “Direct effect” refers to the effect of Attention on Closeness & Liking when Similarity is not included in the model and when it is included in the model (in parentheses).

To learn more about the two distinct facets of group cohesion—one reflecting proximal aspects of group cohesion and the other distal (i.e., symbolic) aspects—we conducted two follow-up mediation analyses [65,66]. With regard to proximal group cohesion, in the negative mood condition, there was a significant indirect effect of attention on Closeness & Liking through Similarity, *b* = .17, 95% BCa CI [.02, .40]. This represents a medium effect, κ^2^ = .13, 95% BCa CI [.02, .30]. The fact that the direct path including Similarity as second predictor fails to be significant indicates full mediation (Fig 6): These results support that similarity is an antecedent to cohesion. Furthermore these results suggest that experiencing negative mood in unison instead of individually increases felt similarity and that the level of similarity in turn predicts closeness and liking.

**Fig 7:**
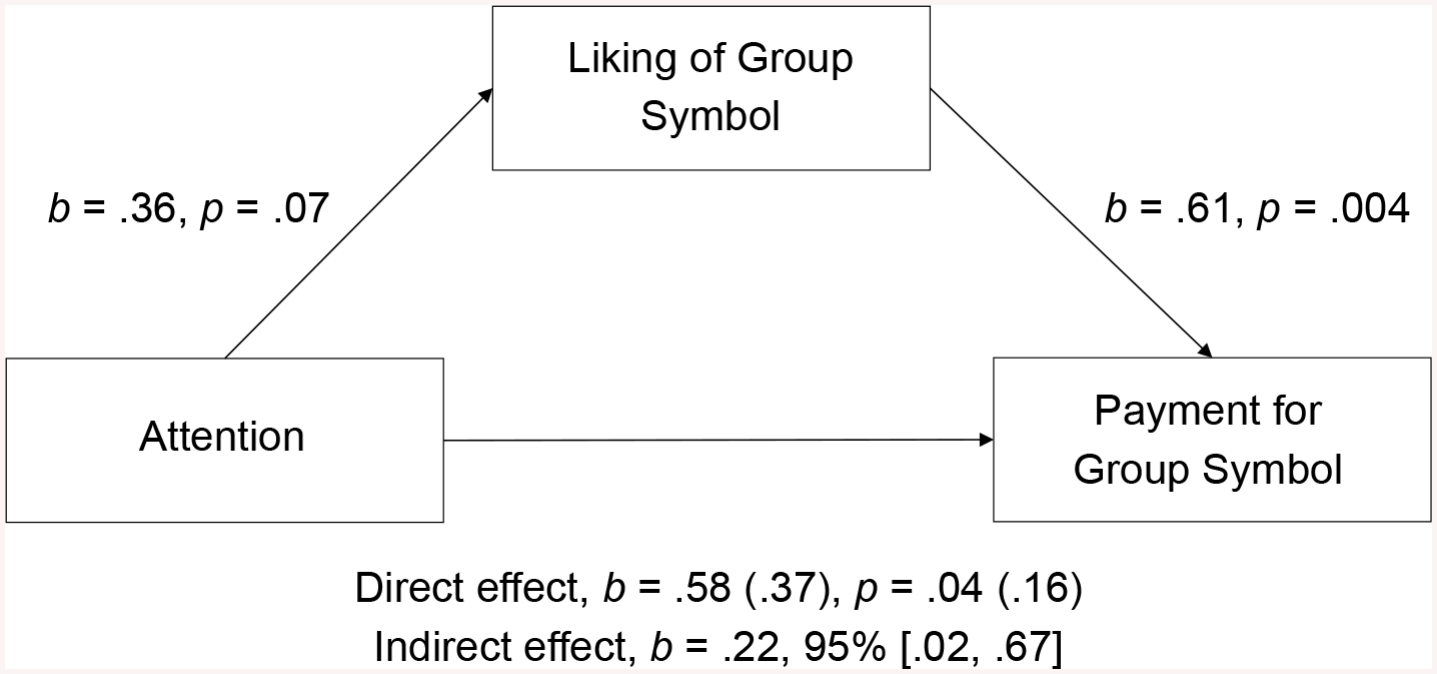
Liking of Group Symbol as mediator. Model of Attention as predictor of Payment for Group Symbol, mediated by Liking of Group Symbol. “Direct effect” refers to the effect of Attention on Payment for Group Symbol when Liking of Group Symbol is not included in the model and when it is included in the model (in parentheses).

With regard to distal group cohesion, the effect of attention on payment for the group symbol was fully mediated by participants’ liking of the symbol, *b* = .22, 95% BCa CI [.02, .67]. This represents a medium effect, κ^2^ = .12, 95% BCa CI [.01, .29]: Participants who attended to the negative film clip in unison liked the group symbol more and consequently spent more money on it (Fig 7).

Summing up, the drop in cohesion after experiencing negative mood individually was compensated when negative mood was experienced in unison. Furthermore, similarity mediated the effect of attention on closeness & liking and liking of group symbol mediated the effect of attention on payment for group symbol.

## Discussion

It is widely believed that collective gatherings are social bonding events. Group cohesion is most important in times of crisis, when mutual support is most needed. This cohesion-enhancing effect of collective gatherings has been argued to stem from emotional synchronization among participants of gatherings. However, prior research has not fully answered the question if the cohesion-enhancing effect of emotional synchrony is driven by the active sharing of emotional experiences or if shared attention to emotional stimuli is sufficient to spark social bonding. This study sought to contribute to a better understanding of emotional synchrony by investigating the latter process. Specifically, we addressed the question if shared attention fosters cohesion compared to individual attention, when participants do not have the opportunity to express their experiences to those around them. We predicted any cohesion-enhancing differences between shared and individual attention to be most pronounced when participants attend to highly arousing negative stimuli rather than non-negative stimuli.

In line with this buffer-hypothesis of shared attention, participants who attended to an arousing negative film clip individually reported a lower level of similarity as well as closeness and liking towards their co-participants, liked a group symbol less and paid less for a group symbol compared to participants who shared attention to this arousing negative film clip. In other words, the cohesion-reducing effect of intensely negative mood was buffered when participants experienced it in unison with their co-participants. Mediation analyses supported the view that the beneficial effect of shared attention on closeness and liking is driven by a shift in perceived similarity to co-participants. Likewise, the beneficial effect of shared attention on payment for a group symbol was driven by a shift in liking this group symbol. The fact that shared attention led to the same pattern of results concerning these two facets of social cohesion speaks to the robustness of our findings: While similarity as well as closeness and liking assess the proximal effect of shared attention and bear on the mental representation of social connectedness, liking of and payment for a group symbol reflect a more distal, implicit outcome of cohesion and refer to a symbolic object.

As expected, the effects of shared attention we found in this study were smaller than those of active emotional sharing. Across four studies, Páez et al. [8] reported medium to large effects with weighted average correlation coefficients of r = .56 between emotional synchrony and identity fusion and of r = .34 between emotional synchrony and social integration, whereas in the study at hand we found small effects (with η_P_^2^ = .02 for all four dependent variables, when comparing individual attention with shared attention to intense negative stimuli).

Given the low intensity of our treatment (i.e., four minutes of watching a film clip together), the smaller effect is not surprising. Furthermore, prior research found that existing social bonds foster the synchronization of emotional reactions [67] and that the effect of interpersonal synchrony in turn is most pronounced when coupled with shared intentionality [68]. As in this study participants did not know each other and did not share a common goal, the fact that we still observed a beneficial effect of shared attention provides tentative evidence of an independent effect of shared attention on group cohesion. Lastly, the current study was the first attempt at a new paradigm. By fine-tuning the experimental manipulation and/or increasing the intensity of the manipulation, future studies may observe stronger effects than were found here.

### Theoretical Implications

The results of the current study have several theoretical implications for research on emotional sharing. First and foremost, we provide evidence that it is not only the process of actively sharing intense negative affect and disclosing one’s emotions to others that fosters unity, but that the mere act of jointly focusing attention on the same intensely negative content is able to elicit the effect. Furthermore, the results of our study offer new insights concerning the construct of I-sharing [21], which is defined as an identical subjective experience in reaction to a stimulus, by suggesting that a synchronous experience of intensely negative affect can bring about group cohesion without the existence of overt cues (e.g., visible reactions) through relying solely on inferred synchrony.

Our results also align with Nummenmaa et al. [30,33], who found evidence for highly arousing negative affect to most effectively synchronize brain activity among participants and concluding that this synchronization sets the mental ground for social bonding to evolve. The results of the current study directly connect to this conclusion, as we used the same manipulation (mood induction through video clips), but directly assessed social cohesion rather than brain activity. Taking together the results of the two studies, there is much to suggest that it is indeed the across-person synchronization of brain activation that causes the bonding effect of experiencing intense negative emotions in unison.

Our results also inform research on emotional priming by demonstrating that the effect of mood induction is contingent on the context in which emotions are experienced, especially when investigating the evaluation of social stimuli. According to Rimé [14] the experience and regulation of emotions is an interpersonal process from early on. This study supports this view by showing that an intense negative affect polarizes when experienced together instead of individually.

Lastly, the study at hand connects back to early research on collective emotions or collective effervescence by Durkheim that was taken up by Collins [69–71], who declared shared attention the eye of the needle through which the power of collective gatherings must pass. As Páez et al. [8] remark, the proposition of Durkheim and his successors has remained untested to this date. With this study we corroborate one core aspect of this influential theory by providing preliminary evidence for the impact of shared attention on social cohesion.

### Strengths, Limitations, and Future Research

A strong point of this experiment is the assessment of several facets of the outcome measure group cohesion and related constructs. By including diverse scales, which addressed different manifestations of the construct (i.e., affective and cognitive) and by assessing proximal (e.g., felt closeness), as well as distal effects (e.g., payment for a group symbol), we were able to more comprehensively understand the effects of shared attention. Moreover, the implementation of self-report scales, both verbal and visual, as well as a behavioral measure, reduced the probability of common-method bias to occur. Furthermore, by using an experimental design, we were able to establish causality and achieve high internal validity. To also secure high external validity, future research should replicate our findings in a field setting to substantiate the generalizability of conclusions.

Speaking to the robustness of our results, the cohesion-enhancing effect of shared attention was observed beyond the effect of common identity—participants in all conditions were assigned a shared social category by wearing tags sporting the group symbol. Thus, the observed effect of shared attention was strong enough to exceed the minimal group paradigm (e.g., [55]). This being said, the possibility remains to be tested that the cohesion-enhancing effect of shared attention is contingent on there being some common identity in the first place. There is some evidence, that prior social bonds are the prerequisite for shared attention to elicit synchronization of emotional reactions [67]. Specifically, future studies should examine whether shared attention calls forth more or less cohesion if there is no induction of common identity prior to the manipulation of attention, unlike in the present study where common identity was induced as a general backdrop. Based on the data at hand we cannot rule out the possibility that common identity is a precondition for shared attention’s cohesion-enhancing effect.

A limitation of this study is the lack of a more direct measure of emotional synchrony. Due to the manipulation check and prior studies on mood induction through film clips [30,33], we can be confident in assuming that participants experienced similar affect while watching the movie clips. However, it remains unclear if the participants were aware of the synchronized affective experience in their group and if awareness of synchrony is a prerequisite for an impact on cohesion. The reason why we abstained from investigating affective synchrony more directly in the current study is twofold: First, research on mimicry found that the effect of mimicry on rapport vanishes if the participants are aware of the imitation [72]. We suspected that a direct measure of affective synchrony (e.g., by asking participants if they believed to share the same emotional experience) would raise awareness and thus diminish the potential effect on group cohesion. While such a restriction was not found for behavioral synchrony [56], we do not know at this early stage of investigation if emotional synchrony does or does not have to go unnoticed to be effective. Second, the main purpose of the current experiment was to investigate the general effect of shared attention on group cohesion. Considering the fact that this field is largely unstudied, we believe that it is most fruitful to first establish the general effect of shared attention on cohesion before investigating the underlying mechanisms.

Another limitation pertains to the fact that we elicited affective states using films, which naturally come with different contents. In other words, the film clips we used not only varied in arousal and valence but also in content. Consequently, our conclusion that changes in group cohesion are solely attributable to the type of arousal and valence could be and should be challenged in future experiments. To prevent the occurrence of a strong priming effect through content, we composed the film clips from different movies with different contents. Furthermore, the highly arousing negative film clip, which increased group cohesion in the shared attention condition, was deliberately selected to not touch on the concept of group cohesion or groups in general. Thus, we believe that it is unlikely that the observed effects on group cohesion are solely due to the content of the film clips. In general, laboratory research on emotions faces the problem of reliably inducing emotions that are as similar as possible across participants while differentiating as clearly as possible across conditions. Films are one of the most commonly used materials in eliciting emotions [54] because of their comparatively high ecological validity [73] as well as their effectiveness in eliciting affective states [64]. Thus, by using film clips, we have relied on a state-of-the-art emotion elicitation procedure.

In conclusion, the current experiment is a first step in investigating the independent effect of passive emotional sharing, and we see one of the main assets of this study in channeling and inspiring future research. Specifically, we encourage research on the following issues:

1. To shed light on the mechanisms of the effects reported in this study, future studies might illuminate the underlying process from an objective as well as from a subjective perspective. Concerning the objective perspective, future research might consider neuropsychological changes that accompany affective synchrony [30] and link them to the extent of cohesion, like what has already been done for the impact of behavioral synchrony on prosocial behavior [74]. Concerning the subjective perspective, a fruitful path for future research might be to investigate which inner processes (e.g., motives, needs, thoughts) drive the effect of shared attention on social bonding. A worthwhile starting point for this research endeavor is the finding that people share positive and negative affect to the same extent, however for distinct reasons—positive emotions are shared to capitalize on the positive emotional state, while negative emotions are shared to gain cognitive clarity and reduce stress [75].
2. Future studies are needed to directly compare the effect of passive emotional sharing (i.e., shared attention to the same emotional stimuli, as examined in this study) versus active emotional sharing (i.e., interpersonal interaction such as talking, weeping, laughing) on group cohesion. Besides a comparison of the magnitude of the effect, it needs to be investigated if passive and active emotional sharing instigate the same processes that ultimately lead to an increase in group cohesion. Although in this study we provided preliminary evidence that a shift in similarity contributed to the increase in closeness, in the case of active emotional sharing the mutual dyadic influence due to an interpersonal interaction might unleash different and/or additional processes.
3. As started in this study, future research may more closely investigate the relationship between different facets of group cohesion. As expected, the proximal and distal facets of cohesion used in our study were only mildly correlated. Yet, we observed the same pattern of results across these facets, which increases our confidence in the robustness of the effect. A fruitful way for future studies may be to test hypotheses concerning the relationship between these facets and/or to investigate if the effect of shared attention extends to larger groups or even organizations as a whole. For example, the finding that shared attention enhanced liking of and payment for the group symbol might reflect heightened devotion to or identification with the company as a whole (Salt + Pepper) that was introduced in the cover story. As participants did not have any information about the company other than its corporate design, but they had their concrete “colleagues” who were part of the company surrounding them, we believe this facet more likely reflects lower-level group identification rather than higher-level organizational identification. However, in the end, the group is contained in the organization, and the organization makes the group an entity. At any rate, this level-of-effect issue points to the fact that it is highly practically relevant to explore how shared attention affects manifestations of higher-level cohesion such as organizational identification or organizational commitment.
4. In this study the individual was the unit of investigation. Future studies could complement this research by inquiring into the effect of emotional experiences on cohesion from a group perspective. For example, research on collective emotional climate discusses that emotional sharing of negative collective events can elicit solidarity and a negative emotional climate at the same time [75]. In this regard, it seems promising to further analyze the specific implications of group-based versus collective emotions. While group-based emotions refer to the emotional experience of an individual in response to group-related events, collective emotions prelude to the perception of what most members of a collective feel in a given situation. In most instances, both types of emotions coincide (e.g., the soccer fan feels elated, because her team won the match, and she perceives that the other fans share this feeling). In some instances, however, they differ (e.g., a wrongdoing of the group suggests a specific emotional reaction, such as guilt, to be appropriate; however, most group members do not feel that way) [76]. Future studies could investigate the interplay of group-based versus collective emotions regarding their effect on group cohesion.

### Practical Implications

Our study provides initial evidence that experiencing intense negative emotions in unison with others buffers the otherwise detrimental effect of negative mood on social cohesion. The fact that a minimal treatment as in this study (four minutes of shared attention) can make a difference gives a foretaste of the impact of big interventions (e.g., involving many people and/or longer stretches of shared attention) on its participants and the social groups to which they belong. Consequently, our research informs the dealing with situations when the experience of negative affect is inevitable (e.g., the closing of an organization, lay-offs or the death of a leader). Our study suggests that in these situations it is advisable to capitalize on the buffering effect of shared attention. Instead of informing those affected individually (as seen in the example of Dell Computers and Radio Shack mentioned in the introduction), managers should communicate bad news in a social setting, as the fact that shared attention can affect cohesion without any interaction taking place points to the applicability of shared attention as a tool to larger groups and/or instances when interaction is difficult (e.g., virtual teams).

## Conclusion

It has been argued that shared affect in groups is evolutionarily significant as it facilitates group bonding [6]. While prior research focused on the role of active emotional sharing on group cohesion; to our knowledge, this experiment is the first to establish causality between shared attention and group cohesion. Our study reveals that sharing attention to intense negative affect for only a few minutes prevents the otherwise cohesion-reducing effect of intense negative affect. These findings suggest capitalizing on the buffer effect of shared attention in times of crisis.
